# Increases in cardiac vagal modulation following muscle mechanoreflex activation via passive calf stretch: Impact of interindividual differences

**DOI:** 10.1113/EP092498

**Published:** 2025-05-11

**Authors:** Georgia C. S. Lehnen, Marcela S. Araujo, Igor S. Rocha, Jeann L. Sabino‐Carvalho, Rosa V. D. Guerrero, Gabriel S. Trajano, Lauro C. Vianna

**Affiliations:** ^1^ School of Exercise and Nutrition Sciences Queensland University of Technology Brisbane Australia; ^2^ NeuroV̇ASQ̇ ‐ Integrative Physiology Laboratory, Faculty of Physical Education University of Brasilia Brasilia Brazil; ^3^ Division of Renal Medicine, Department of Medicine Emory University School of Medicine Atlanta Georgia USA

**Keywords:** autonomic nervous system, exercise pressor reflex, mechanoreflex, muscle stretching exercises

## Abstract

Muscle mechanoreflex is crucial to cardiac vagal modulation during exercise and can be activated during passive calf stretch. Herein, we aimed to determine whether cardiac vagal modulation following a single session of passive stretch is linked to interindividual cardiac vagal responses at the onset of passive calf muscle stretching in healthy young adults. Twenty‐four volunteers (10 women) completed the experimental conditions in a randomised order over different days: a time‐control condition and five sets of 1 min of unilateral passive stretching of the calf, with 15 s of rest between each stretching trial. Heart rate and systolic and diastolic blood pressure were continuously measured on a beat‐to‐beat basis before, immediately following, and at 15 and 30 min after the passive stretching intervention. Interindividual variations in cardiac vagal inhibition during the passive stretching session were identified, classifying volunteers into responder (*n* = 16) and non‐responder (*n* = 8) groups. The onset of passive muscle stretching elicited an immediate reduction in cardiac vagal modulation (*P* = 0.026) and an increase in heart rate (*P* = 0.009) for the responders only. Cardiac vagal modulation significantly increased following 30 min of passive stretching (*P* = 0.010 vs. rest) for the responders only. During time control, all cardiac vagal variables were unchanged for both groups. In summary, our findings demonstrate that a single session of passive calf muscle stretching can enhance cardiac vagal modulation, but this effect is dependent on interindividual responses at the onset of stretching. These results highlight the role of muscle mechanoreflex activation in cardiac autonomic regulation and suggest that passive stretching may have potential cardiovascular benefits, particularly for individuals who exhibit a mechanoreflex‐mediated response.

## Abstract (highlights)

- **What is the central question of this study?**
  Muscle mechanoreflex is crucial to cardiac vagal modulation during exercise and can be activated during passive calf stretch: is cardiac vagal modulation following a single session of passive stretch linked to interindividual cardiac vagal responses at the onset of passive calf muscle stretching?
- **What is the main finding and its importance?**
  A single session of passive calf muscle stretching can enhance cardiac vagal modulation, but this effect is dependent on interindividual responses at the onset of stretching. These results highlight the role of muscle mechanoreflex activation in autonomic regulation and suggest that passive stretching may have potential cardiovascular benefits.

## 1 INTRODUCTION

Autonomic control of cardiovascular function during exercise is primarily regulated by two key neural mechanisms: central command and the exercise pressor reflex. Central command is a feed‐forward mechanism that activates cardiovascular and somatomotor systems through signals originating in higher brain centres (Raven et al., 2002; Vianna et al., 2009; Williamson et al., 2006). In parallel, the exercise pressor reflex functions as a feedback mechanism, triggered by the activation of mechano‐ and metabosensitive afferents within skeletal muscles (Cui et al., 2008; Kaufman & Hayes, 2002; Mitchell, 2013). Both mechanisms modulate autonomic outflow, orchestrating the interplay between the sympathetic and parasympathetic nervous systems, which contributes to heart rate and blood pressure responses in an intensity‐dependent manner (Teixeira et al., 2020). Central command and the exercise pressor reflex, particularly mediated by muscle mechanoreflex (involving primarily group III fibres in skeletal muscle), are posited to significantly contribute to cardiac vagal withdrawal during the transition from rest to exercise (Cui et al., 2006; Drew et al., 2008; Gladwell et al., 2005; Kaufman & Hayes, 2002; Teixeira et al., 2018; Teixeira & Vianna, 2022; Vianna et al., 2018, 2010; Williamson et al., 1985). This mechanism is critical for facilitating rapid cardiovascular adjustments that enhance oxygen delivery to the exercising muscles (Vianna et al., 2010; Wray et al., 2005). Conversely, the contribution of the muscle metaboreflex to vagal withdrawal, primarily mediated by group IV fibres in skeletal muscle, is believed to be minimal at the onset of exercise (Cui et al., 2006; Fisher et al., 2015; Kaufman & Hayes, 2002; Vianna et al., 2008).

Animal studies indicate that activating mechanoreceptors through passive stretching results in increased heart rate and blood pressure, along with decreased cardiac vagal activity and increased sympathetic outflow (Murata & Matsukawa, 2001; Stebbins et al., 1988; Wilson et al., 1994). Similarly, in humans, pharmacological inhibition of parasympathetic activity via intravenous glycopyrrolate results in a marked reduction in the chronotropic response to passive stretching (Gladwell et al., 2005), suggesting that muscle mechanoreceptors are implicated in cardiac vagal modulation at the onset of exercise. Importantly, studies involving both humans and animals have shown that cardiac vagal changes during muscle mechanoreflex activation depend, in part, on central GABAergic mechanisms. This suggests that skeletal muscle afferents could be a significant source of synaptic drive to GABAergic neurons within the nucleus tractus solitarius (NTS) (Potts et al., 2003; Teixeira et al., 2018). Nevertheless, the cardiovascular response to muscle mechanoreflex tends to be transient and of small magnitude (Cui et al., 2006; Cui et al., 2008; Drew et al., 2008; Teixeira et al., 2018; Vianna et al., 2018), and the mechanisms underlying these responses are not fully understood.

Despite extensive research investigating the cardiovascular responses to passive calf muscle stretching (Baum et al., 1995; Gladwell et al., 2005; Gladwell & Coote, 2002; Inami et al., 2014), findings remain inconsistent. Baum et al. (1995) observed a progressive increase in blood pressure during sustained stretching without concomitant changes in heart rate. In contrast, Gladwell et al. (2005) provided evidence that passive stretching induces a vagally mediated increase in heart rate despite no significant changes in blood pressure. These discrepancies suggest that passive stretching may elicit distinct autonomic responses among individuals, warranting further investigation into potential underlying mechanisms. Given the uncertainty surrounding the cardiac autonomic responses to passive stretching, interindividual variability may be a crucial factor influencing cardiovascular effects. While previous studies have reported distinct patterns in blood pressure and heart rate responses, whether passive stretching differentially modulates cardiac vagal activity across individuals remains unclear. In support of this possibility, previous research has identified distinct autonomic responder profiles, with some individuals exhibiting increases and others decreases in muscle sympathetic nerve activity following physiological stressors, even in homogeneous samples of healthy young adults (Donadio et al., 2002; Incognito et al., 2018; Teixeira & Millar, 2025). Unlike earlier studies that analysed mean group data, classifying individuals based on their cardiac autonomic responses may provide new insights into cardiac vagal regulation following muscle mechanoreflex stimulation, such as passive stretching.

Interindividual variability in cardiac autonomic responses to stretching may help explain differences in post‐exercise adaptations. However, whether cardiac vagal responses to muscle mechanoreflex activation during passive calf muscle stretching influence vagal modulation following stretching remains unclear, as limited studies (Gladwell & Coote, 2002; Inami et al., 2014) have monitored this period, and those that have, only assessed very short recovery times. Furthermore, despite extensive documentation of the numerous benefits of active stretching, such as reductions in stress hormones, improved mental states, reductions in muscular tone, increased flexibility, and vascular adaptations (Eda et al., 2020; Mueck‐Weymann et al., 2004; Nóbrega et al., 2005; Poole et al., 1997; Shinno et al., 2017; Wong & Figueroa, 2019), the potential of passive stretching to increase cardiac vagal modulation following a single session remains acutely unclear. This is important because previous studies suggesting improvements in cardiac autonomic function following a stretching session were not designed to isolate the contribution of muscle mechanoreflex from central command or metaboreflex activation (Farinatti et al., 2011; Fisher et al., 2005; Mueck‐Weymann et al., 2004; Oliver et al., 2023; Shinno et al., 2017). This approach would allow us to gain valuable mechanistic insights into how stretching may influence cardiac vagal modulation following the session, with potential implications for optimizing exercise protocols and improving cardiovascular health.

Thus, the present study aims to determine whether cardiac vagal modulation following a single session of passive stretch is linked to interindividual responses at the onset of passive calf muscle stretching in healthy young adults. We hypothesise that: (1) there will be significant interindividual variability in cardiac vagal responses to mechanoreflex activation; (2) cardiac vagal modulation will increase following passive calf muscle stretching; and (3) the magnitude of increase in cardiac vagal modulation after a single session will be associated with the extent of cardiac vagal withdrawal during passive calf muscle stretching.

## 2 METHODS

### 2.1 Ethical approval

All study procedures were approved by the Ethics Committee of the University of Brasília (CAAE: 85088924.3.0000.5558) and conducted in accordance with the latest revision of the *Declaration of Helsinki*, except for registration in a database. Written informed consent was obtained from all participants prior to their inclusion in the study.

### 2.2 Participants

Twenty‐four healthy (10 women), normotensive, non‐smoker volunteers who were physically active for at least 3 months (self‐reported, 3 days per week) participated in this study. None of the volunteers used controlled medications or had a cardiopulmonary, neurological or metabolic disease history. All volunteers were instructed to refrain from caffeine and alcohol consumption, as well as from engaging in physical exercise or stretching sessions, for 24 h prior to the tests.

### 2.3 Experimental protocols

All testing was conducted within a temperature‐controlled environment (∼24°C). The volunteers attended the laboratory three times, scheduled at the same time of day, with sessions separated by a minimum of 24 h and a maximum of 48 h. Volunteers were familiarised with the equipment and procedural protocols during the initial familiarization session. Additionally, the maximum tolerable passive torque during calf stretching was measured by passively dorsiflexing the foot to an angle just below the threshold of discomfort reported by the volunteer, thus applying a load deemed to produce a sensation of ‘appropriate stretching’. Volunteers were explicitly instructed not to experience pain, and any trial in which pain was reported would result in exclusion from the study.

**FIGURE 1:**
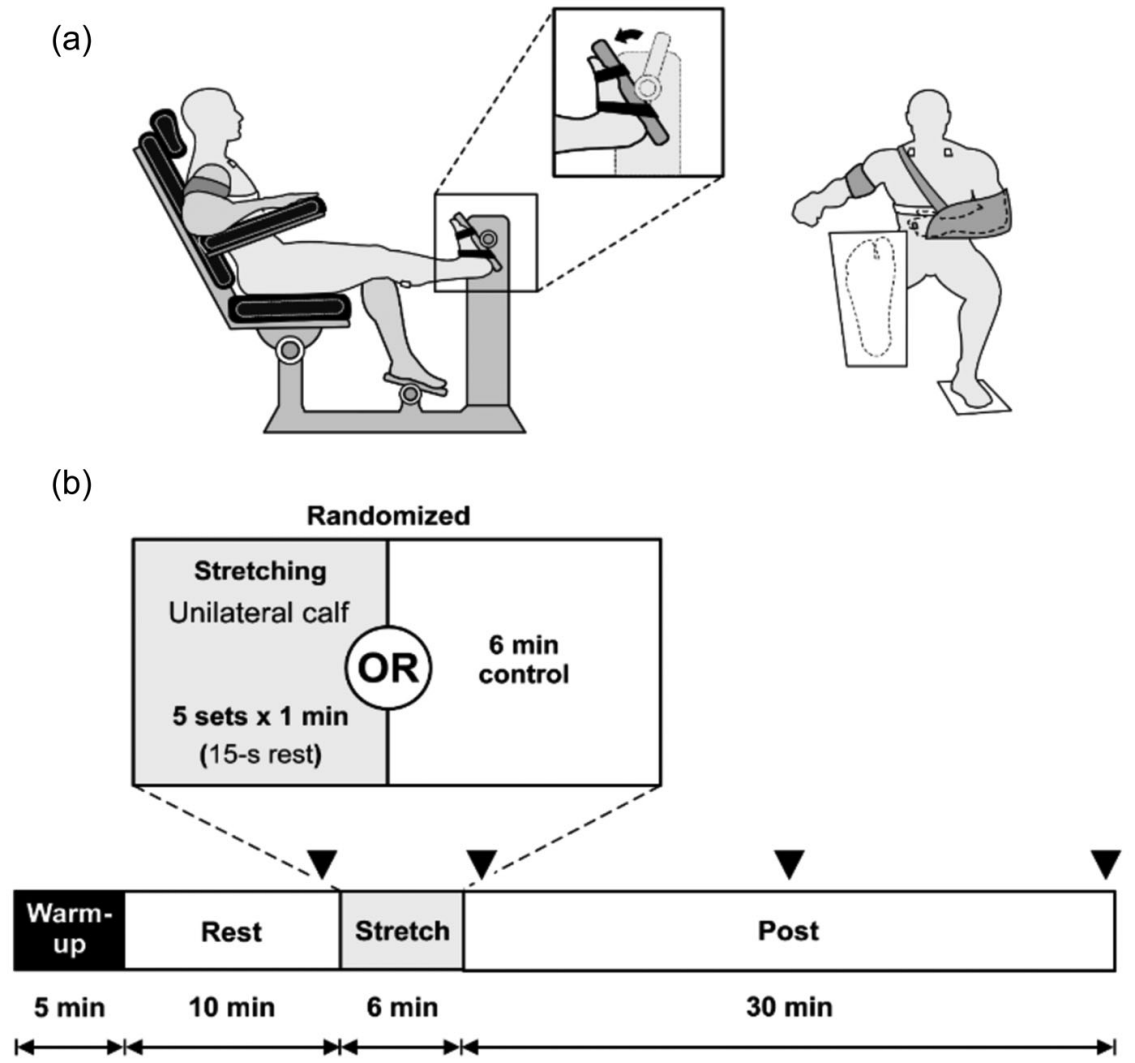
(a) Lateral and front view of the volunteer position during the stretching protocol. (b) Schematic representation of the experimental protocol employed in the present study. ▼ represents the time when the cardiorespiratory and autonomic variables were analysed: immediately before, immediately after and 15 and 30 min post‐intervention for both passive stretching and the control condition.

In the subsequent two sessions, which constituted the experimental trials, volunteers engaged in a 5‐min warm‐up on a stationary bike, following a 2–3 Borg Rating of Perceived Exertion. Following the instrumentation process, volunteers were instructed to sit comfortably and rest for 10 min to stabilise cardiovascular parameters. Subsequently, participants completed the following experimental conditions in a randomised order across different days: (1) control (no stretch – 6 min of rest) and (2) five sets of 1 min of unilateral passive stretching of the calf, interspersed with 15 s of rest (Figure 1). The stretching protocol was chosen based on previous studies that employed similar methodologies to investigate the effects of passive stretching on autonomic and neuromuscular responses (Drew et al., 2008; Trajano et al., 2014).

The passive muscle stretching was performed using an isokinetic dynamometer (Biodex System 4, Biodex Medical Systems, Shirley, NY, USA). While seated at a 115° hip flexion angle and 0° knee angle, the right calf was subjected to dorsiflexion at a rate of 5° s⁻¹ until passive resistance reached 90% of the maximal tolerable stretch torque, as determined during the familiarization session. The apparatus included a metal plate designed for foot placement, complete with heel support and Velcro straps to secure the foot in position. Adjustments to the heel's position and the foot's height were made to ensure a consistent rotation point for each participant. The left leg remained relaxed on the chair. The joint angle was continuously adjusted towards dorsiflexion during the stretch to maintain the initial passive torque value.

### 2.4 Measurements

Heart rate, along with systolic and diastolic blood pressure, was continuously measured on a beat‐to‐beat basis utilizing Lead II electrocardiography (MSC‐6111, CardioMatic, New York, NY, USA) and finger photoplethysmography (Human NIBP Controller, ADInstruments, Bella Vista, NSW, Australia) positioned on the middle finger of the non‐dominant hand. The resting absolute beat‐to‐beat blood pressure values were calibrated using concomitant brachial arterial blood pressure measurements obtained via an automated digital sphygmomanometer (Dixtal DX2022, Manaus, AM, Brazil) to confirm the validity of finger measurements. Specifically, the average of three brachial measurements was taken during the resting period to calibrate the finger pressure, and additional measurements were performed at time points following the stretching intervention. Respiratory frequency was monitored using a pneumatic belt securely positioned around the abdomen (MLT1132 Piezo Respiratory Belt Transducer, ADInstruments) to mitigate the potential confounding effects of substantial respiratory excursions on cardiovascular measurements (e.g. the Valsalva manoeuvre).

To ensure the absence of muscle contractions during passive stretching, electromyographic activity was recorded from the plantar flexors of the same limb using a Bagnoli‐2 EMG System (Delsys, Natick, MA, USA), employing two silver‐coated electrodes, 10 mm in diameter and spaced 15 mm apart, placed on the muscle belly. A reference electrode was positioned on the same limb. The electromyography (EMG) signals were subsequently amplified and filtered. All continuous data were collected using LabChart (version 8; PowerLab, ADInstruments).

### 2.5 Cardiac vagal indices

Cardiac vagal modulation in both control and experimental conditions was assessed following the 5‐min recording guidelines established by the Task Force on Heart Rate Variability (Heart Rate Variability, 1996) to calculate average cardiovascular variables at rest and during recovery. These parameters were evaluated immediately before, immediately after, and at 15 and 30 min post‐intervention for both conditions (Figure 1). Additionally, to assess the immediate cardiac vagal modulation to passive stretching, a 15‐s window at the onset of stretching was used, as this time frame captures the transient activation of the muscle mechanoreflex, as demonstrated in previous studies (Gladwell & Coote, 2002; Oliver et al., 2023; Vianna et al., 2010).

Ectopic beats detected in the electrocardiogram traces were identified through both automated and manual methods before exclusion from the analysis. Three volunteers were excluded based on this criterion, and only segments free from interference were analysed (Heart Rate Variability, 1996). Cardiac vagal modulation was quantified using linear methods in the time and frequency domains. In the time domain, we assessed the root mean square of successive RR interval differences (RMSSD) and the percentage of adjacent NN intervals differing by more than 50 ms (pNN50), which reflect cardiac vagal activity. In the frequency domain, we analysed high‐frequency power (HF, 0.15–0.4 Hz), which is widely accepted as an indicator of parasympathetic modulation. All analyses were conducted using SinusCor (v1.0.2, Rio de Janeiro, RJ, Brazil).

Volunteers were categorised as responders or non‐responders based on changes in RMSSD. A responder was identified when the value during stretching was lower than the pre‐stretching RMSSD (i.e. RMSSD_stretching_ – RMSSD_pre<0_), indicating inhibition of cardiac vagal modulation. RMSSD was chosen as the primary classification criterion because it is a well‐established time‐domain measure of short‐term cardiac vagal modulation, unlike pNN50 and HF power, which require longer time windows for reliable estimation (Bartels et al., 2017).

Given its central role in short‐term blood pressure regulation, cardiac baroreflex function was assessed as an additional indicator of cardiac vagal modulation. The baroreflex buffers transient blood pressure fluctuations via rapid heart rate adjustments, and its gain is widely accepted as a proxy for parasympathetic activity (Parati et al., 2000). Beat‐to‐beat time series of systolic blood pressure (SBP) and RR intervals were analysed using the sequence technique for estimating spontaneous cardiac baroreflex sensitivity (cBRS) (CardioSeries v2.4, Sao Paulo, SP, Brazil), as previously described (Antonino et al., 2017; Parati et al., 2000; Sabino‐Carvalho et al., 2020, 2018; Samora et al., 2019). The sequence technique identifies consecutive beats in which progressive increases in SBP are followed by a progressive lengthening in the RR interval, or vice versa. Arterial baroreflex sequences were detected only when at least three consecutive cardiac cycles exhibited a corresponding increase or decrease in both SBP (≥1 mmHg) and RR interval (≥1.0 ms). A linear regression was applied to each individual sequence, and only those with *R* ^2^ > 0.85 were included in the analysis. The slope of the SBP–RR interval relationship was then averaged across all identified sequences, providing an estimate of spontaneous cBRS gain, which reflects the magnitude of the cardiac vagal response to spontaneous fluctuations in blood pressure.

### 2.6 Statistical analysis

The normality of the data distribution was assessed using the Shapiro–Wilk test. Variables following a normal distribution were analysed using parametric tests, while non‐normally distributed variables were analysed using non‐parametric methods.

For between‐group comparisons, an independent sample Student's *t*‐test was applied for normally distributed variables, and the Mann–Whitney *U*‐test was used as a non‐parametric alternative when the normality assumption was not met. For within‐group comparisons, a repeated measures ANOVA or Friedman's test was employed to evaluate time‐dependent changes, followed by Wilcoxon's *post hoc* test with Bonferroni's correction for multiple comparisons.

To evaluate within‐subject reproducibility, the intraclass correlation coefficient (ICC) was calculated using a two‐way mixed model, absolute agreement. The ICC was computed separately for RMSSD, pNN50 and HF in responders and non‐responders to determine test–retest reliability across all time points, with values interpreted as excellent reliability (ICC > 0.90), good reliability (ICC = 0.75–0.90), moderate reliability (ICC = 0.50–0.75) and poor reliability (ICC < 0.50).

A significance level of *P* < 0.05 was established for all analyses. All statistical analyses were performed using SPSS Statistics version 20 for Windows (IBM Corp., Armonk, NY, USA).

## 3 RESULTS

Table 1 outlines the baseline characteristics of the volunteers, demonstrating that responders and non‐responders were matched for age, body mass, height, body mass index (BMI), torque and position during the stretching intervention (maximum value).

**TABLE 1:** Participants characteristics.

Variable	Responders (*n* = 16)	Non‐responders (*n* = 8)	*P*
Age, years	22 ± 3	23 ± 2	0.378
Body mass, kg	73 ± 15	71 ± 12	0.836
Height, m	1.71 ± 0.1	1.74 ± 0.1	0.508
BMI, kg/m^2^	25 ± 3	23 ± 2	0.345
Torque, N m	27.82 ± 10.20	34.32 ± 7.54	0.126
Position, °	18.9 ± 2.9	20.5 ± 2.0	0.165

Values are represented as means ± SD. Abbreviation: BMI, body mass index.

**FIGURE 2:**
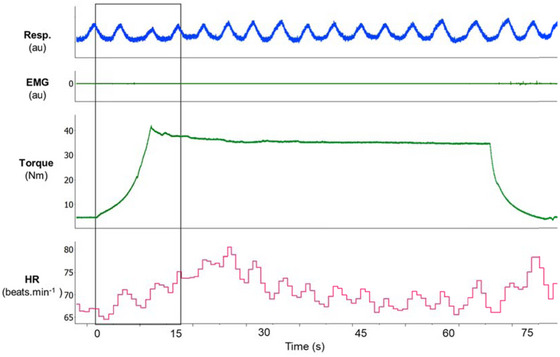
Original recording showing a respiratory signal, a muscle EMG signal, a typical torque trace and a heart rate at the onset of passive muscle stretching.

Figure 2 presents a recording of the respiratory belt, muscle EMG signal, typical torque trace and heart rate, highlighting the onset of passive muscle stretching, which lasted 15 s.

Confirming that passive stretching did not elicit muscle activation, RMS EMG showed no significant differences between conditions for responders (rest: 0.081 ± 0.028 mV vs. stretching: 0.082 ± 0.029 mV, *P* = 0.642) or non‐responders (rest: 0.101 ± 0.034 mV vs. stretching: 0.101 ± 0.032 mV, *P* = 1.000). Additionally, no differences were found between responders and non‐responders at rest (*P* = 0.178) or during stretching (*P* = 0.126).

**FIGURE 3:**
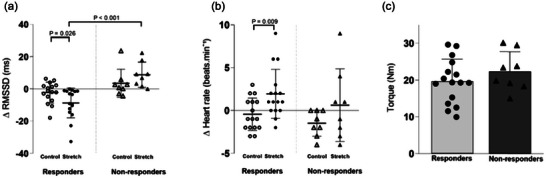
(a, b) Changes (∆) in RMSSD (a), and changes in heart rate (b) between control (open symbols) and stretching (filled symbols) conditions for responders (circles, *n* = 16) and non‐responders (triangles, *n* = 8). Changes in RMSSD and heart rate represent the difference between the control condition (first 15 s of the 6‐min set) and pre‐control (rest), and between the stretching condition (average of the first 15 s of the onset of each of the five sets) and pre‐stretching (rest). (c) Mean torque values during the first 15 s of each of the five sets for responders (circles) and non‐responders (triangles), with filled symbols indicating the stretching condition.

Figure 3a,b presents the changes (∆) in RMSSD and heart rate during the control condition and stretching protocol. A total of 16 participants (seven women) were classified as responders, while eight participants (three women) were categorised as non‐responders. Specifically, responders exhibited a significant reduction in RMSSD (−8.8 ± 9.3 ms), indicating cardiac vagal withdrawal during stretching, whereas non‐responders did not show a reduction (10.6 ± 9.4 ms) (Figure 3a). Fisher's exact test revealed no significant difference in sex distribution between groups (*P* = 1.000), indicating that sex was not a confounding factor in the observed cardiac autonomic responses.

The RMSSD response to passive muscle stretching differed between responders and non‐responders, confirming distinct group cardiac vagal response patterns. The between‐group difference was significant (*P* < 0.001), while no significant difference was observed under the control condition (*P* = 0.178; Figure 3a). Similarly, heart rate responses during stretching did not differ significantly between groups (*P* = 0.228; Figure 3b). When comparing the changes induced by passive stretching versus the control condition within each group, a significant reduction in RMSSD was observed in responders (*P* = 0.026; Figure 3a), accompanied by a significant increase in heart rate (*P* = 0.009; Figure 3b). However, no significant differences were found in RMSSD (*P* = 0.208; Figure 3a) or heart rate (*P* = 0.231, Figure 3b) in non‐responders. It is important to mention that torque measurements remained consistent across both responder and non‐responder groups (*P* = 0.292; Figure 3c).

Table 2 summarises the measured haemodynamic variables. No significant differences were observed between groups at rest for the following variables: respiratory frequency (*P* = 0.643), heart rate (*P* = 0.927), systolic blood pressure (*P* = 0.219), diastolic blood pressure (*P* = 0.759) and mean blood pressure (*P* = 0.645). Furthermore, no significant differences persisted for these variables immediately after stretching, 15 min post‐stretching and 30 min post‐stretching. However, a significant difference for RMSSD in responders (*P* = 0.023) was found. *Post hoc* Wilcoxon tests, with Bonferroni correction (*P* = 0.016), confirmed a significant increase in RMSSD at 30 min post‐stretching compared to rest (*P* = 0.010), while no significant differences were observed between rest and immediately after stretching (*P* = 0.477) or rest and 15 min post‐stretching (*P* = 0.093). For pNN50, no significant changes were detected over time (*P* = 0.248), but an increasing trend was observed (mean rank: rest, 2.13; post‐stretching, 2.31; 15 min, 2.56; 30 min, 3.00) for responders. Similarly, HF did not reach statistical significance (*P* = 0.075) but showed an upward trend (mean rank: rest, 1.88; post‐stretching, 2.38; 15 min, 2.75; 30 min, 3.00). In non‐responders, none of the cardiac vagal modulation indices exhibited significant changes over time (RMSSD: *P* = 0.896, pNN50: *P* = 0.522, HF: *P* = 0.985). Furthermore, RMSSD, pNN50 and HF remained stable across all time points during the control condition, with no significant differences between responders and non‐responders (all *P* > 0.05).

**TABLE 2:** Haemodynamic variables at rest (pre‐stretching), immediately after stretching (post‐stretching), and 15 and 30 min after the stretching protocol for responders (*n* = 16) and non‐responders (*n* = 8).

	Respiratory frequency, breaths/min	Heart rate, beats/min	Systolic BP, mmHg	Diastolic BP, mmHg	Mean BP, mmHg	RMSSD, ms	pNN50, %	HF, ms^2^
Rest								
Responders	17 ± 3	71 ± 6	108 ± 12	59 ± 6	75 ± 8	56.3 ± 22	33.5 ± 17	1511.1 ± 1074
Non‐responders	18 ± 4	70 ± 9	113 ± 8	59 ± 6	76 ± 7	48.0 ± 21	31.4 ± 23	878.7 ± 704
Post‐stretching								
Responders	16 ± 3	69 ± 5	110 ± 11	60 ± 8	77 ± 8	59.8 ± 31	35.0 ± 22	1959.0 ± 1771
Non‐responders	17 ± 4	70 ± 10	113 ± 8	61 ± 7	78 ± 8	49.2 ± 24	31.2 ± 24	1012.4 ± 897
15 min post‐stretching								
Responders	16 ± 3	69 ± 5	110 ± 12	59 ± 9	76 ± 9	63.3 ± 33	37.0 ± 20	2112.6 ± 2175
Non‐responders	18 ± 5	69 ± 9	113 ± 8	60 ± 7	77 ± 8	49.4 ± 26	30.4 ± 24	1066.9 ± 1007
30 min post‐stretching								
Responders	15 ± 3	69 ± 6	109 ± 12	61 ± 8	77 ± 8	65.8 ± 33*	39.0 ± 21	2207.9 ± 2034
Non‐responders	18 ± 4	69 ± 9	113 ± 10	62 ± 9	79 ± 9	50.6 ± 26	29.7 ± 23	1089.9 ± 1076

Values are expressed as means ± SD. *P < 0.05 versus rest (pre‐stretching). Abbreviations: BP, blood pressure; HF, high‐frequency; pNN50, percentage of RR intervals differing by more than 50 ms; RMSSD, root mean square of successive RR interval differences.

To assess the within‐subject reproducibility of vagal indices, the ICC was calculated based on repeated measures across all participants. ICC values for average measures were 0.967 for RMSSD (95% CI: 0.938–0.984), 0.980 for pNN50 (95% CI: 0.964–0.991), and 0.932 for HF (95% CI: 0.873–0.967), all with *P* < 0.001, indicating excellent reliability.

**FIGURE 4:**
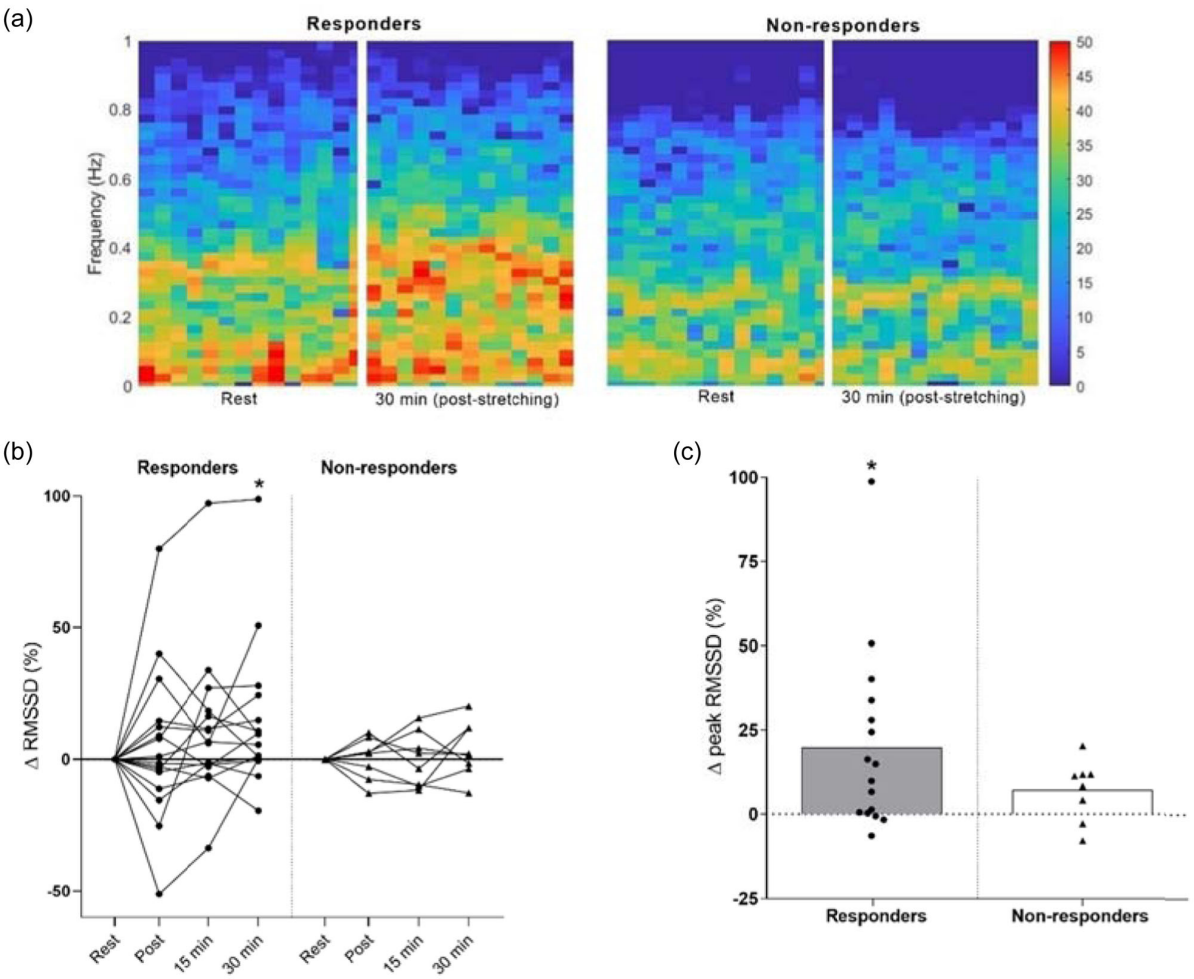
(a) Scaled heat maps representing frequency‐domain data for responders (left) and non‐responders (right) at rest (pre‐stretching) and 30 min post‐stretching. (b) Percentage change (∆%) in RMSSD at post‐stretching time points (immediate post, 15 and 30 min), with individual values represented by circles (responders, *n* = 16) and triangles (non‐responders, *n* = 8). (c) Individual peak RMSSD responses. *P< 0.05 versus rest (pre‐stretching).

Figure 4 illustrates the frequency and time‐domain responses to passive stretching, emphasizing both the overall spectral patterns and individual variability. The scaled heat maps display representative frequency‐domain distributions from one responder and one non‐responder, captured at rest (pre‐stretching) and 30 min after the intervention (Figure 4a). These visualizations highlight distinct autonomic modulation profiles between individuals, reinforcing the presence of heterogeneous cardiac vagal responses to passive stretching. In the time domain, the percentage change (Δ%) in RMSSD increased during the recovery period (immediate post, 15 and 30 min) in responders, with a significant increase at 30 min compared to rest (*P* = 0.017; Figure 4b). Additionally, the individual peak value of RMSSD was only significantly different from zero for responders (*P* = 0.005; Figure 4c). Furthermore, RMSSD (Δ%) during the control period indicated that cardiac modulation remained unchanged between the rest and recovery period for both groups (responders: *P* = 0.631; non‐responders: *P* = 0.798).

Resting spontaneous cardiac baroreflex sensitivity (cBRS) was comparable between responders and non‐responders (19.4 ± 7.1 vs. 18.2 ± 8.9 ms mmHg⁻¹, *P* = 0.721). Additionally, cBRS values remained stable during the recovery period for both groups: immediately after stretching (19.5 ± 9.4 vs. 18.2 ± 8.7 ms mmHg⁻¹, *P* = 0.747), at 15 min (19.6 ± 7.1 vs. 18.3 ± 11.7 ms mmHg⁻¹, *P* = 0.749) and at 30 min post‐stretching (20.4 ± 9.0 vs. 19.5 ± 11.5 ms mmHg⁻¹, *P* = 0.837).

## 4 DISCUSSION

This study is the first to examine the impact of interindividual variability in cardiac vagal responses to muscle mechanoreflex activation on cardiac vagal modulation during 30 min of recovery period. Our findings provide significant insights into this relationship. Firstly, in alignment with our hypothesis, we observed a notable decrease in cardiac vagal activity and a concomitant increase in heart rate upon the initiation of passive muscle stretching, particularly within a group of responders (16 responders compared to 8 non‐responders). This highlights a substantial interindividual variation in cardiac vagal responses to passive muscle stretching. Secondly, the responders demonstrated a marked improvement in cardiac vagal modulation 30 min following the passive calf muscle stretching. These results shed light on the link between enhanced cardiac vagal modulation after passive stretching and the variability in individual responses to the stretching stimulus. Importantly, our findings reveal that the decrease in cardiac vagal activity – indicated by a reduction in RMSSD – coupled with an increase in heart rate, occurs particularly among the responders. This significant interindividual variability underscores the necessity of considering individual differences when analysing cardiac autonomic responses to passive muscle stretching.

Considering these findings, the results of this study are consistent with the established framework of the exercise pressor reflex (Drew et al., 2008; Gladwell & Coote, 2002; Nóbrega et al., 1993; Teixeira et al., 2018; Vianna et al., 2010; Williamson et al., 1985), which posits that the initiation of a physical stimulus, such as passive muscle stretching, induces a withdrawal of parasympathetic tone and an accompanying elevation in sympathetic nervous system activity, ultimately leading to an increase in heart rate. Specifically, the activation of group III mechanoreceptors, which are responsive to mechanical deformation of skeletal muscle, is likely responsible for mediating the observed alterations in heart rate during passive stretching (Teixeira et al., 2018; Vianna et al., 2010). In contrast, group IV fibres, which typically respond to metabolic changes such as chemical signals and muscle fatigue, are not believed to significantly contribute during passive stretching due to the stable conditions within the muscle at that time (Fisher et al., 2015; Vianna et al., 2008). Moreover, the design of the present study mitigates the potential influence of central command, defined as the brain's volitional component (Nóbrega et al., 1993; Vianna et al., 2009). This distinction is of paramount importance, as it indicates that the cardiac responses to passive stretching are largely governed by the activation of skeletal muscle mechanoreceptors, thereby contributing to a more comprehensive understanding of the physiological mechanisms involved.

No prior studies have investigated the cardiac vagal inhibition response at the onset of mechanoreflex activation through passive muscle stretch in humans, specifically distinguishing responders from non‐responders. The mechanisms underlying the variability in cardiac vagal inhibition, characterised by a decrease in RMSSD, during the initial phase of passive calf muscle stretch, remain unclear. However, differences in activation or sensitivity of the exercise pressor reflex among individuals may contribute to this variability. Indeed, between‐subject differences in the activation or gain of the exercise pressor reflex have been proposed as a potential mechanism underlying variability in muscle sympathetic nerve activity responses (Incognito et al., 2018). These insights have significant implications for future research and practical physical therapy and sports science applications. Tailoring stretching protocols to accommodate individual autonomic differences could enhance therapeutic and performance outcomes.

The time course of the cardiac vagal modulation after a single stretching session presented here is consistent with previous findings within the literature (Farinatti et al., 2011; Inami et al., 2014). For example, Farinatti et al. (2011) examined participants with low flexibility, who performed three sets of 30‐s active static stretches targeting the hamstrings and trunk. Their use of a Polar R‐R monitor to record various cardiac vagal modulation metrics revealed increased parasympathetic activity 30 min post‐stretching. A key distinction in our study is the assessment of passive stretching, which effectively isolates the involvement of muscle mechanoreflex from central command. This approach allows us to gain valuable mechanistic insights into how stretching may influence the autonomic nervous system (Williamson et al., 1985). Supporting our findings, other studies examining chronic whole‐body stretching have reported acute changes in autonomic regulation after stretching (Eda et al., 2020; Mueck‐Weymann et al., 2004; Patil et al., 2015; Shinno et al., 2017), consistently indicating parasympathetic dominance and decreased sympathetic activity. The mechanisms by which muscle mechanoreceptors increase cardiac vagal modulation after stretching are not fully understood. However, our previous findings showing GABAergic modulation of mechanoreflex‐induced changes in cardiac vagal modulation (Teixeira et al., 2018), combined with animal studies exploring the interaction between substance P and GABA in the NTS (Chen & Bonham, 2010), provides a compelling framework for understanding the observed increase in cardiac vagal modulation after passive calf stretches. Specifically, passive calf stretching activates mechanoreceptors that send signals to the NTS, potentially resulting in a less intense release of substance P compared to more vigorous exercise involving larger muscle mass. This activation may still influence GABAergic activity within the NTS, leading to increased vagal tone, similar to the autonomic shift seen in post‐exercise hypotension, albeit on a smaller scale. Additionally, it is possible that the magnitude of vagal modulation positively correlates with the extent of mechanoreflex activation, influencing the NTS‐mediated cascade. However, this hypothesis requires further investigation.

This study investigated the effects of passive calf muscle stretch on post‐stretch cBRS (i.e. employing the sequence technique), finding no significant effect despite an observed increase in parasympathetic activity 30 min post‐stretching. The dissociation between the increase in RMSSD and the unchanged cBRS observed in this study may be attributed to the distinct physiological processes these measures reflect. RMSSD primarily captures high‐frequency fluctuations in heart rate associated with respiratory sinus arrhythmia and is considered a sensitive index of rapid, beat‐to‐beat parasympathetic modulation (Heart Rate Variability, 1996). In contrast, cBRS, as assessed by the sequence technique, reflects the reflexive ability of the cardiovascular system to adjust heart rate in response to spontaneous changes in blood pressure, which may be influenced by both parasympathetic tone and baroreflex gain itself (Sabino‐Carvalho et al., 2021). Supporting this idea, Hesse et al. (2007) demonstrated that changes in heart rate variability account for only 10% of the variance in cardiac baroreflex sensitivity, indicating a weak association between these variables. Furthermore, it is possible that passive stretching increased tonic vagal modulation (as reflected in RMSSD), without affecting the reflexive responsiveness of the baroreflex pathway. Additionally, the sequence method used to assess cBRS may be less sensitive to subtle, tonic shifts in vagal activity compared to heart rate variability indices. Previous studies have also suggested that baroreflex sensitivity is more likely to be influenced when mechanoreflex activation is accompanied by metabolic stress, which was absent in the current protocol (Cui et al., 2008; Drew et al., 2008). Thus, the increase in cardiac vagal modulation after passive stretching may occur independently of baroreflex adjustments, highlighting that these two measures, although related, do not always change in parallel.

### 4.1 Limitations

The generalizability of the present findings is constrained by several methodological limitations. Firstly, the study population consisted exclusively of healthy young adults, precluding direct extrapolation of the results to older individuals or those with pre‐existing cardiovascular conditions, where both autonomic and mechanoreflex function may be significantly compromised. Secondly, while a frequently employed method, the passive calf muscle stretch protocol may not fully recapitulate the physiological complexity of mechanoreflex activation observed during more dynamic exercise settings or when involving other muscle groups. The potential contribution of metabolically induced sensitization of muscle afferents, a factor implicated in modulating mechanoreflex responsiveness in other studies, was not directly investigated in this study. Finally, the employed sequence technique for baroreflex sensitivity assessment differs from alternative approaches, such as the neck chamber method, potentially introducing variability in comparative analyses with existing literature. These limitations underscore the need for future investigations employing diverse participant populations and incorporating more comprehensive assessments of both mechanoreflex activation and autonomic function under a broader range of physiological conditions.

### 4.2 Future directions

This study highlights a significant acute effect of a single passive stretching session on cardiac autonomic control, specifically demonstrating an increase in parasympathetic activity as measured by RMSSD. While regular stretching is known to improve flexibility (Nóbrega et al., 2005), the immediate impact of a single session on parasympathetic tone is a novel finding. This insight may have practical implications for exercise routines and cardiovascular health. In addition to its acute effects, regular stretching can help improve vascular function and potentially benefit the autonomic nervous system (Wong & Figueroa, 2019). Further studies are warranted to test this possibility.

### 4.3 Conclusion

In conclusion, there is substantial interindividual variability in cardiac vagal responses to passive calf muscle stretching (i.e. mechanoreflex activation), as demonstrated by the classification of 16 responders and 8 non‐responders. Cardiac vagal modulation increased 30 min post‐stretching in responders but remained unchanged in non‐responders. Furthermore, the magnitude of post‐stretching increases in cardiac vagal modulation was associated with the extent of cardiac vagal withdrawal during stretching, reinforcing the link between acute mechanoreflex activation and subsequent cardiac autonomic responses. These findings highlight the importance of considering interindividual differences when evaluating the cardiac autonomic effects of passive stretching and suggest that the initial cardiac vagal response to mechanoreflex activation may influence longer‐term cardiac autonomic outcomes.

## AUTHOR CONTRIBUTIONS

Georgia C. S. Lehnen, Gabriel S. Trajano and Lauro C. Vianna conceived and designed the study. Georgia C. S. Lehnen, Marcela S. Araujo, Igor S. Rocha and Lauro C. Vianna performed the experiments. Georgia C. S. Lehnen, Marcela S. Araujo, Jeann L. Sabino‐Carvalho, Rosa V. D. Guerrero and Lauro C. Vianna analysed the data. Georgia C. S. Lehnen, Gabriel S. Trajano and Lauro C. Vianna interpretated the data. Georgia C. S. Lehnen and Lauro C. Vianna designed the figures. Georgia C. S. Lehnen, Gabriel S. Trajano and Lauro C. Vianna drafted the paper. Georgia C. S. Lehnen, Marcela S. Araujo, Igor S. Rocha, Jeann L. Sabino‐Carvalho, Rosa V. D. Guerrero, Gabriel S. Trajano and Lauro C. Vianna edited and revised the manuscript. All authors have read and approved the final version of this manuscript and agree to be accountable for all aspects of the work in ensuring that questions related to the accuracy or integrity of any part of the work are appropriately investigated and resolved. All persons designated as authors qualify for authorship, and all those who qualify for authorship are listed.

## CONFLICT OF INTEREST

The authors have nothing to report.

## Data Availability

The data underlying our findings can be shared upon reasonable request directed at the corresponding author.
